# Effect of Nonisoprene Degradation and Naturally Occurring Network during Maturation on the Properties of Natural Rubber

**DOI:** 10.3390/polym14112180

**Published:** 2022-05-27

**Authors:** Guojing Chen, Bingbing Wang, Hongtu Lin, Wenfeng Peng, Fuquan Zhang, Gaorong Li, Dongbin Ke, Jianhe Liao, Lusheng Liao

**Affiliations:** 1 School of Materials Science and Engineering, Hainan University, Haikou 570228, China; chengj0909@163.com (G.C.); wangbbhn@163.com (B.W.); dobit123@163.com (D.K.); 2 Key Laboratory of Tropical Crop Products Processing of Ministry of Agriculture and Rural Affairs, Agricultural Products Processing Research Institute of Chinese Academy of Tropical Agricultural Sciences, Zhanjiang 524002, China; zjoulht@163.com (H.L.); pwfpwx@163.com (W.P.); xqcyl2106@163.com (F.Z.); ligaorong0509@163.com (G.L.); 3 Hainan Provincial Key Laboratory of Natural Rubber Processing, Zhanjiang 524002, China

**Keywords:** natural rubber, maturation, naturally occurring network, nonisoprene degradation, mechanical property

## Abstract

It well-known that the superior performance of natural rubber (NR) compared to its synthetic counterpart mainly derives from nonisoprene components and naturally occurring network, which varies during the progress of the maturation and thereby results in technically graded rubber with different properties. However, identifying the roles of these two factors in the forming of excellent performance of NR is still a challenge as they change simultaneously during the maturation process. Here, influences of naturally occurring networking and nonisoprene degradation on the components, structures and properties of NR were systematically investigated by tailored treatments of maturation. It was found that the maturation-induced formation of natural network structure contributes to the increase in initial plastic value, Mooney viscosity and gel content for un-crosslinked NR, while the decomposition of nonisoprene components plays a dominant role in improving the mechanical properties of vulcanized NR. Stress-strain curve and Mooney-Rivlin analysis demonstrate that the biodegradation of the nonisoprene components significantly boost the vulcanization process, which significantly increases the number of chemical cross-link networks and effective cross-link density of the material, greatly improving the mechanical properties of NR vulcanizates. This resulted in the tensile strength of TSR 10CV being able to reach 22.6 MPa, which is significantly improved compared to 15.8 MPa of TSR 3CV. Evidenced by tubular model fitting, the increase in chemical cross-linking points effectively reduces the movable radius of the molecular chain under dynamic loading, making the molecular chain more difficult to move, which suppresses the entropy change under dynamic loading and consequently endows NR excellent dynamic mechanical properties. This resulted in a significant decrease in the temperature rising of TSR 10CV to 3.3 °C, while the temperature rising of TSR 3CV was still as high as 14.5 °C. As a minor factor, the naturally occurring network improves the mechanical properties of vulcanizates in the form of sacrificial bonds.

## 1. Introduction

Natural rubber (NR) is a kind of biomacromolecule fabricated by collecting, coagulating and drying a colloidal suspension from Hevea Brasiliense, demonstrating excellent comprehensive properties, such as high mechanical performance, low heat build-up, good wear resistance, high flexibility at low temperature and high adhesion to steel cord [1,2]. Due to obvious superiorities to its counterparts, NR has been employed in versatile solutions to satisfy the demanding requirements in the characteristics of high-performance, production efficiency, sustainability, etc. [3,4,5,6]. Nowadays, NR is an increasingly important elastomeric material for our daily life applications spanning many domains of military, chemistry, hygiene and medical sectors. It is estimated that there are nearly 1000 kinds of plants producing polyisoprene in the world, but NR from Hevea brasiliensis trees are emerging as the most promising in practical usage and large-scale fabrication. However, the yield of NR that is seriously restricted by land resources and natural conditions has become the fatal bottleneck for its extensive application. Therefore, unraveling and tuning the structure-associated performance of NR is a significant issue in industry and academia.

Compared with synthetic polyisoprene that is a linear chain resulting from the polymerization of monomer, bio-produced NR possesses more complex architectures which are critical in determining its comprehensive properties that could not be expected from a simple combination of the individual components [7]. It is well-known that NR consists of approximately 94% cis-1, 4-polyisoprene and 6% nonisoprene components (such as proteins, phospholipids, etc.) [8], which generates a complex network structure and is defined as “naturally occurring network” [1,9]. Evidenced by rheological response and dielectric relaxation results, such naturally occurring network in NR was confirmed by Huang and Guo et al. [10,11]. Comparison investigation by Toki et al. demonstrates that the presence of a naturally occurring network structure formed by natural components in un-vulcanized NR significantly facilitates strain-induced crystallization and enhances modulus and tensile strength [12]. For vulcanized NR, there will be three types of networks in vulcanized NR, i.e., vulcanized cross-linked network, naturally occurring network and entanglements, where naturally occurring network and entanglements contribute to higher effective crosslink densities and stronger strain-induced crystallization, respectively [13]. At the same time, the nonisoprene components in NR not only directly participate in the construction of the naturally occurring network, but also can promote the vulcanization reaction of NR; this facilitates the formation of a more complete vulcanized cross-linked network [14]. Wei et al. demonstrated by differential scanning calorimetry that the introduction of nonisoprene components can effectively reduce the activation energy of the vulcanization reaction and increase the mechanical property of the vulcanizate [15]. In addition, some nonisoprene components such as protein also can act as antioxidants in NR and consequently enhance the heat resistance of vulcanizates and un-vulcanizates NR [16]. The above results indicate that the unique naturally occurring network and nonisoprene components are the key factors for the excellent performance of NR.

It is recognized that the desirable properties of NR are not only dependent on the hierarchical architecture but also on the fabrication process. Briefly, the manufacture of NR involves bioproduction, collection, coagulation, maturation and drying of natural rubber latex, where biocomponents as well as natural occurring network change significantly [17]. As a bioproduct of Hevea brasiliensis tress, the mesostructured and nonisoprene content of NR can be tuned by collecting NRL from different clones [16,18]. The macromolecular structure also changed after tree opening, while the weight-average molecular weight increased and stabilized after 7.5 months of tapping, the bimodal molar mass distribution primarily involved short chains at the opening of the trees [19]. At the same time, the choice of coagulation method will also directly affect the content of non-isoprene components in the NR [20]. Usually, compared with naturally coagulated, acid-based coagulation endows NR higher nonisoprene components. Moreover, there are a large number of active microorganisms in NRL, which will influence the content of nonisoprene components and the naturally occurring network during maturation due to their own metabolic reactions [21,22,23]. Considering that the microorganisms in the NRL have good activity, maturation actually occurs in all stages before drying of the fabrication of natural rubber, which plays a significant role in determining the properties of rustled NR materials [24,25,26]. In order to further amplify the microorganism-based maturation process, some NR grumes are always stocked for several weeks, which is commonly adapted in the manufacture of technically specified rubber (TSR) [21]. With higher plasticity, resilience and wear resistance, TSR has been proved to be an alternative to tire production, accounting for about 70% of the raw material market. Therefore, the maturation process plays an important role in regulating the properties of NR. However, the evolution of biocomponents-associated natural architecture during maturation and its influence on the performance of resulted NR materials is unclear.

**Scheme 1:**
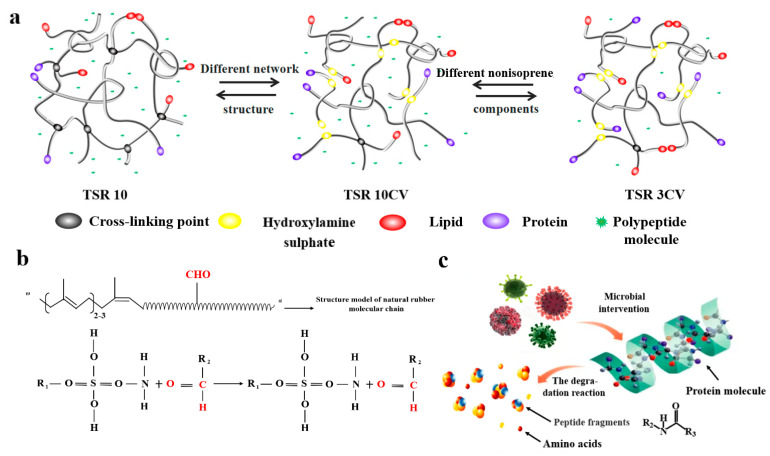
(**a**) Schematic description of naturally occurring network transformation and degradation of nonisoprene components; (**b**) Mechanisms by which hydroxylamine sulfate prevents the formation of naturally occurring networks; (**c**) Nonisoprene components are degraded by microorganisms.

Thus, three samples of Technically Specified Rubber 10 (TSR 10), Technically Specified Rubber 10 Constant Viscosity (TSR 10CV) and Technically Specified Rubber 3 Constant Viscosity (TSR 3CV) were prepared to distinguish the effects of the complex maturation process. At first, we prepare No.1 sample TSR 10 which undergoes a complete maturation process without any chemical treatment, in which the naturally occurring network structure and nonisoprene components have both changed. No. 2 Sample TSR 10CV was matured for the same duration as TSR 10 to ensure the same microbial degradation reaction as TSR 10. In addition, hydroxylamine sulfate was added to Sample No. 2 to limit the formation of its naturally occurring network during the maturation process. The purpose is to make these two samples have the following properties: after maturation, the same degree of decomposition of nonisoprene components and different degrees of naturally occurring network structure. By comparing these two samples to reflect the effect of the naturally occurring network present during the maturation process on the performance. Subsequently, we introduced hydroxylamine sulfate to No. 3 sample TSR 3CV to make it have a similar degree of naturally occurring network structure as sample No. 2, and directly added formic acid to solidify sample No. 3 to prevent the reaction of microbial degradation. The purpose is to make Samples No. 2 and No. 3 have the following properties: the same degree of naturally occurring network structure and different degrees of decomposition effect of nonisoprene components after maturation, and by comparing the two samples to reflect the effect of the decomposition of nonisoprene components on the properties during the maturation process. Based on the experimental design described above, we prepared Scheme 1. In Figure a of Scheme 1, we mainly describe the possible situations of the natural network structure and nonisoprene components within the three samples after experimental adjustment, and introduce the differences between the three samples by comparing them with each other. In Figure b of Scheme 1, we mainly describe the principle of hydroxylamine sulfate for inhibiting the formation of the naturally occurring network structure in NR. In Figure c of Scheme 1, we mainly describe the degradation process of nonisoprene components in NR by microorganisms and the introduction of possible products. This work is the first to differentiate the effects of nonisoprene component decomposition and naturally occurring network on NR performance during maturation. At the same time, in addition to testing some conventional mechanical properties such as tensile strength, we especially compared and analyzed the dynamic mechanical properties that users care about and are close to daily use; this has a certain practical guiding role for better regulation of NR production and processing means in the future.

## 2. Materials and Methods

### 2.1. Materials

Natural rubber latex used in the present work was fresh nature rubber latex with 33.7% dry rubber content (without ammonia) and provided by Shuguang Farm of Gaozhou Nongken Group Company (Guangzhou, China). Zinc oxide (ZnO), stearic acid (SA), sulfur (S) and 2-mercaptobenzothiazole (accelerator M) were purchased from Zhanjiang Keming Co., Ltd. (Hangzhou, China) Formic acid (analytical purity), toluene (analytical purity), hydroxylamine sulfate (analytical purity) and tetrahydrofuran (THF, HPLC grade) were purchased from Shanghai Aladdin Biochemical Technology Co., Ltd. (Shanghai, China).

### 2.2. Preparation of TSR10, TSR10CV and TSR3CV

**Scheme 2:**
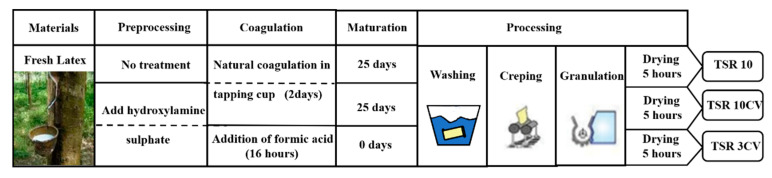
Schematic diagram of the preparation process of TSR 10, TSR 10CV, TSR 3CV.

Fresh nature rubber latex with 33.7% dry rubber content (without ammonia) was used in this experiment. Each NR sample was made from the latex of a single clone (IAN873) in order to remove variability linked to the clonal origin of the latex. The trees were opened for tapping in 1998 and had been in exploitation for 20 years. According to local industrial recommendations, the trees were stimulated by ethephon (a concentration of 3.0% to 3.5%) and tapped once every 15 days on a half spiral. The fresh latex collected on the same day was diluted and then divided into 3 parts. The specific schematic diagram is shown in Scheme 2 and the fabrication details are as follows:

(1) Technically Specified Rubber 10 (TSR 10): The diluted latex was left to coagulate naturally in the cups. The coagula were retrieved after 5 days, and deposited on barns for an additional 21 days. Then the coagula were finally prepared into the samples by washing, crushing, creping, granulating and drying for 5 h at 110 °C.
(2) Technically Specified Rubber 10 Constant Viscosity (TSR 10CV): 0.8 wt.‰ of final dry rubber weight Hydroxylamine sulphate was introduced into the diluted latex for 30 min to stabilize the rubber by inhibiting branching between polyisoprene chains. The latex was left to coagulate naturally in the cups for 5 days. Then, the coagula were retrieved and deposited on barns for an additional 21 days. The samples were finally obtained by washing, crushing, creping, granulating and drying the coagula for 5 h at 110 °C.
(3) Technically Specified Rubber 3 Constant Viscosity (TSR 3CV): 0.8 wt.‰ of final dry rubber weight neutral hydroxylamine sulphate was introduced into the diluted latex for 30 min. As a comparison, TSR 3CV does not use a maturation process and the latex was directly coagulated by adjust pH value of solution to 5.4 with 2 wt.%. Then, the sample was stored at room temperature for 20 h. The final TSR 3CV sample was obtained by the same coagulation and drying process as TSR 10 and TSR 10CV samples.

### 2.3. Preparation of NR Vulcanizates

Three different types of natural rubber (TSR 10, TSR 10CV and TSR 3CV) were used to prepare gum according to GB/T 15340, with the formulations shown in Table 1. Firstly, the rubber sample was loaded into a two-roll mill (KY-3220D-160, Kaiyan Machinery Technology Co., Ltd., Dongguan, China) and plasticized for 4 min. Then, the vulcanization ingredient (ZnO, Stearic acid, sulfur, accelerator M) was added into the sample and mixed for another 7 min at 67 °C. Finally, the rubber compound was sheeted out by a two-roll mill and adjusted for at least 24 h at room temperature before testing. Curing properties of the rubber compounds were characterized with a rheometer (MDR-2000E, Liyuan Electronic Chemical Equipment Co., Ltd., Wuxi, China), and the vulcanization of NR sheets were completed at 143 °C for the optimum cure time (tc90) by a compression molding machine (300T700, Hailite Rubber & Plastic Machinery Co., Ltd., Nantong, China).

### 2.4. Characterizations

**Plasticity value.** The method used for the plasticity of NR determination was determined according to the standard of ISO 2007. The test is done by Wallace Rapid Plastometer (P14, Wallace Company, London, UK). Five cylindrical specimens of uniform thickness from the homogenized sample were cut, and taken the median value of the final result. The storage hardening data (Δ*P*) were obtained by measuring the Wallace plasticity of rubber samples with storing for 0 h (*P*_0_) and 24 h (*P_a_*). The condition of accelerating storage hardening reactions was set as 60 °C in an atmosphere dried with phosphorus pentoxide. The plasticity retention index (*PRI*) was obtained by testing the ratio of Wallace plasticity of rubber samples before (*P*_0_) and after (*P*_30_) 0.5 h of constant temperature heating in an oven with constant ventilation (140 °C). The specific calculation equation is as follows (1) and (2):

(1) $$△P=P_{a}-P_{0}$$

(2) $$PRI\left(\%\right)=\frac{P_{30}}{P_{0}}$$

**Gel permeation chromatography.** Gel content of raw rubber was determined according to ISO 2007. The raw rubber (about 0.1 g) was dissolved in tetrahydrofuran (30 mL), and then stored in dark at 25 °C for 20 h without stirring. After being centrifugated at 8000 rpm for 6 h, the supernatant of the sample was filtered through a 0.45 µm filter and diluted with tetrahydrofuran about 1 mg/mL.

The remaining gel fraction was washed with acetone and dried at 110 °C for 1 h in oven after the sol fraction was removed. The gel content was calculated by the following Equation (3):

(3) $$N\left(\%\mathrm{wt}\right)=\frac{m_{a}-m_{0}}{m_{a}}\times 100$$

where *N* is gel content of the sample, *m_a_* and *m*_0_ are the dry weights after extraction and before extraction, respectively.

**Nitrogen contents.** The total nitrogen content of rubber was tested by the Kjeldahl method using a Kjeldahl Apparatus (NKD-6260, Yihong Analytical Instrument Co., Ltd., Shanghai, China) as described in literature [27]. Firstly, 0.5 g of raw NR sample was mixed with 0.2 g CuSO_4_, 3.0 g K_2_SO_4_, 10 mL H_2_SO_4_ (98% mass fraction) and treatment for 3 h. Then, the total nitrogen content of rubber was determined by Kjeldahl Apparatus when the sample cooled down to room temperature. Finally, the nitrogen content was directly obtained by the built-in software of the instrument.

**Fatty acid.** The fatty acid content of rubber was determined with the gas chromatograph (7890A, Agilent Co., Ltd., Santa Clara, CA, USA). The raw NR sample (3.0 g) was mixed with methanol/toluene azeotropic solution (55 mL), pentadecanoic acid solution (1 mL) regarded as internal standard, and 5–10 drops of H_2_SO_4_ solution (98% mass fraction). The mixture solution was reacted and refluxed at 75 °C for 18 h, and then the methyl esterification solution was obtained. The saturated sodium bicarbonate solution (10 mL) and dichloromethane solution (20 mL) were added into the methyl esterification solution for extraction. The extract solution was diluted with dichloromethane (50 mL). Finally, the diluted extract solution was filtered with a 0.45 µm needle-type organic phase filter. The filtrate was injected into a gas chromatograph. In this work, the different chain lengths of fatty acids standards were used to calibrate the columns. The oven temperature was increased from 150 °C to 210 °C at a rate of 2 °C/min for the holding time of 2 min, and then the temperature continued to rise to 230 °C with the rise rate of 50 °C/min for the holding time of 5 min. The retention time and peak area of the different chain lengths fatty acids are directly obtained by the built-in software of the instrument. The equation for calculating the fatty acid content of the sample was as follows (4)–(6):

(4) $$R_{fi}=\frac{A_{n}\times m_{i}}{A_{i}\times m_{n}}$$

where *R_fi_* is response factor of fatty acid methyl ester *i*. *A_n_* and *m_n_* are peak area and weight of internal standard methyl pentadecanoate in the extraction solution of calibration standard mixture, respectively. *A_i_* and *M_i_* are peak area and weight of fatty acid methyl ester *i* in the extraction solution of the calibration standard mixture, respectively.

(5) $$W_{i}=\frac{B_{i}\times m_{n}\times R_{fi}}{B_{n}\times m}\times 100\%$$

(6) $$W=\sum^{\;}W_{i}$$

where *W_i_* is the mass percent of fatty acid *i* in the raw rubber sample, *m_n_* and *B_n_* are weight and peak area of internal standard methyl pentadecanoate in the extraction solution of raw rubber sample, respectively. *B_i_* is peak area of fatty acid methyl ester *i* in raw rubber sample extraction solution. *m*_0_ is the quality of the raw rubber sample. *W* is the total content of fatty acids in the raw rubber sample.

**FT-IR.** Smooth sample with 1mm was used to determine the nonisoprene contents of NR by Fourier Transform Infrared (FTIR) spectrophotometry (Perkin Elmer Inc., Hopkinton, MA, USA) [28]. The samples were scanned 32 times by the FTIR spectra (the wavenumber range from 4000 to 400 cm^−1^) with 4 cm^−1^ resolutions under the attenuated total reflectance (ATR) mode.

**Mechanical properties.** The dumbbell-shaped and right-angle vulcanizates were prepared according to the ISO 527 standard. The tensile strength and tear strength were obtained by the Electro-mechanical Universal Testing Machines (ETM103C, Shenzhen Wance Test Equipment Co., Ltd., Shenzhen, China).

**Dynamic performance.** The columnar vulcanizates were completed by a columnar mould at 143 ℃ in the plate vulcanizing machine (XLB-D, Hongqiao Rubber Machinery Co., Ltd., Huzhou, China). The vulcanization time is *T*_90_ + 10 min.

The fatigue life of vulcanized samples was obtained by the compression fatigue test machine (GABO METER4000, GABO company, Munich, Germany), according to the GB/T 1687.3 standard. The vulcanized sample, obtained from the rubber compounds, was tested for 2000 s at the prestress 1 MPa, stroke 6.35 mm, frequency 30 Hz and 55 °C of thermostatic chamber.

The compression heat generation of vulcanized samples was obtained by the Rubber Compression Heat Generation Analyzer (RHU-2000N, High-speed Railway Testing Instrument Co., Ltd., Dongguan, China) according to the GB/T 1687.3 standard. The vulcanized samples, obtained from the rubber compounds, were measured for 25 min at the prestress 1 MPa, stroke 4.45 mm, frequency 30 Hz and 55 °C of thermostatic chamber.

**Crosslink density tests.** Vulcanized samples with dimensions 10 × 10 × 2 mm^3^ were immersed in toluene solution (15 mL) at 25 °C for a week. Then, wiping the solution attached to the sample surface, the samples were weighed and dried in a vacuum oven at 110 °C for 1 h. The crosslink density of the samples is calculated with the following Equations (7) and (8):

(7) $$V=\frac{-\left[Ln\left(1-\varphi_{p}\right)+\varphi_{p}+x\varphi_{p}{}^{2}\right]}{V_{1}\left(\sqrt[{3}]{\varphi_{p}}-\frac{1}{2}\varphi_{p}\right)}$$

(8) $$\varphi_{p}=\frac{1}{\left[1+\frac{m-md}{md}\right]\frac{\rho_{p}}{\rho_{s}}}$$

where *V*_1_ is the molar volume of toluene, *x* is the Flory-Huggins interaction parameter of toluene and rubber (according to the literature, it is 0.428) [29,30]. *φ_p_* is the volume fraction of rubber in a swollen network. *m* is the mass of the sample after swelling, *md* is the mass of the sample after drying, *ρ_p_* is the density of the polymer, *ρ_s_* is the density of the solvent.

## 3. Results and Discussion

### 3.1. Characterization of Natural Rubber

**Figure 1:**
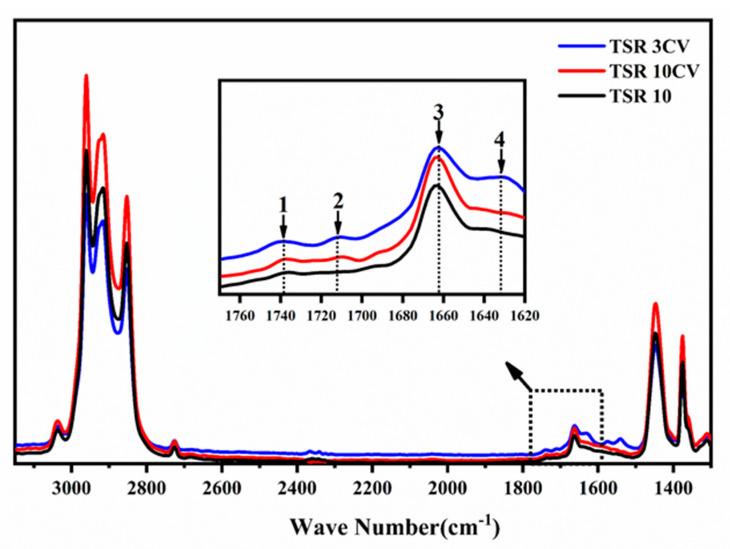
The FT-IR spectra of TSR 10, TSR 10 C, TSR 3CV.

The FT-IR spectra of TSR 10, TSR 10CV and TSR 3CV are shown in Figure 1. It can be clearly seeing that TSR 10, TSR 10CV, and TSR 3CV have similar peak shapes at 3100 to 1800 cm^−1^ and 1500 to 1300 cm^−1^. However, the peak intensity of TSR 3CV at 1740 to 1630 cm^−1^ is significantly higher than that of TSR10CV and TSR10. Among them, the absorption peak at 1630 cm^−1^ points to the N-H stretching vibrations of amide group, and another absorption peak at 1737~1710 cm^−1^ can be related to the lipid stretching vibrations of R_1_ (C = O)-O-R_2_ [31]. This indicates that there are more nonisoprene components in TSR 3CV, which is related to the lack of maturation process. At the same time, these peaks for TSR 10CV and TSR 10 are almost similar, implying that TSR 10 and TSR 10CV had similar degrees of nonisoprene compents due to the same of microbial degradation. In order to more intuitively discuss the content changes of important non-colloidal components in the three samples, the nitrogen content of the samples was tested by Kay apparatus, and the fatty acid content of the samples was tested by gas chromatograph. The specific values are summarized in Table 2. TSR 10 and TSR 10CV had similar nitrogen content and total fatty acid content, while TSR 3CV had significantly higher nitrogen content and total fatty acid content, which was consistent with the trend of FT-IR test.

The Structure parameters of raw rubber for TSR 10, TSR 10CV and TSR 3CV are shown in Table 3. It can be found that TSR10 has the highest gel content, while TSR 10CV and TSR 3CV have a significant decrease in gel content. At the same time, there was a positive correlation between the number of naturally occurring networks and the gel content. Therefore, it is not difficult to see that there are more naturally occurring networks in TSR 10, while the number of naturally occurring networks in TSR 10CV and TSR 3CV is significantly lower. The reason may be that the terminal groups of TSR 10 molecular chains carry out the crosslinking reaction via covalent bonds and hydrogen bonds in the maturation process. Meanwhile, the polyisoprene chains form physical entanglement by van der Waals forces. The above two factors bring about the formation of a naturally occurring network [32]. However, the presence of hydroxylamine sulfate in TSR 10CV and TSR 3CV will passivate the functional groups on the rubber molecular chain, and thus hinder the cross-linking reaction between the rubber molecular chains, which effectively inhibits the formation of a naturally occurring network. In addition, TSR 10 has the highest Mooney viscosity and initial plasticity value, indicating that the natural network structure will improve the difficulty degree of molecular chain in the process of movement, which effectively improves the performance of raw rubber. However, there is little difference between TSR 10CV and TSR 3CV in *P*_0_ and *ML* (1+4), indicating that the decomposition of nonisoprene components has relatively little influence on the performance of raw rubber.

### 3.2. Conventional Mechanical Properties of NR Vulcanizates

**Figure 2:**
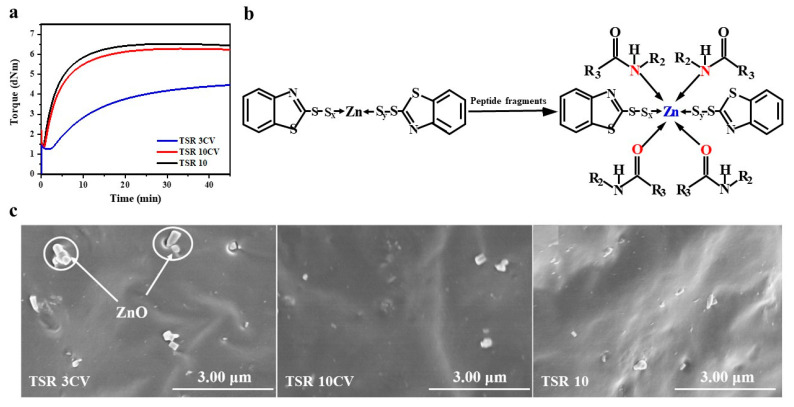
(**a**) Cure curves for TSR 10, TSR 10CV and TSRH 3CV vulcanizates; (**b**) Crosslinking reaction with ZnO [33]; (**c**) SEM images of TSR 3CV, TSR 10CV, TSR 10 (5000×).

Figure 2 shows the cure characteristics of three NR vulcanizates (TSR 10, TSR 10CV, and TSR 3CV). It can be seen that TSR 3CV exhibited lower cure rate index (CRI), maximum torque value (MH) and torque difference, while two samples of TSR 10 and TSR 10CV showed significantly higher CRI, MH and torque difference. This may be due to the nonisoprene components of TSR 10 and TSR 10CV having been degraded by microorganisms during the maturation process. Among them, the protein is mainly decomposed into polypeptide fragments, and the amide bonds on the polypeptide fragments can form coordination interactions with Zn^2+^ ions, which improves the solubility of Zn^2+^ in the matrix [33]. At the same time, with the increase in solubility of Zn^2+^ in the matrix, the energy Ea required for the vulcanization reaction will decrease, which is conducive to the crosslinking reaction. In addition, lipid degradation products also can be used as activators to promote the action of zinc oxide to accelerate the sulfidation reaction [34,35,36]. Therefore, it is not difficult to find that the presence of these degradation products significantly promoted the vulcanization crosslinking reaction of TSR 10 and TSR 10CV. However, proteins, lipids, carbohydrates, and other nonisoprene components in TSR 3CV were retained without the microbial decomposition process, so TSR 3CV vulcanizates showed larger ZnO clusters in SEM testing (Figure 2c), which results in a significant decrease in the effectiveness of the vulcanization reaction. The above results show that the degradation of nonisoprene components during the maturation process is beneficial to promoting the vulcanization reaction. Meanwhile, for TSR 10 and TSR 10CV, the vulcanization characteristics of the two are not much different, which indicates that the naturally occurring network has little effect on the vulcanization process of NR.

**Figure 3:**
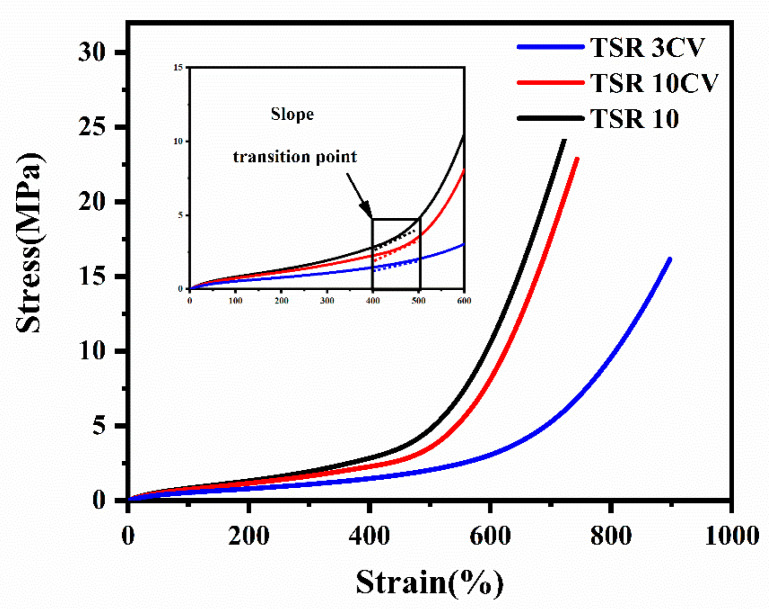
Stress-strain curves for rubber vulcanizates for TSR 10, TSR 10CV and TSR 3CV.

The stress-strain curves of the three vulcanizated samples are compared in Figure 3. It can be found that the stress values of the three samples all increase gradually with the increase in strain, but there are obvious differences in their increasing amplitudes. Among them, the tensile stress of TSR 3CV is the lowest, the stress of TSR 10CV is significantly higher than that of TSR 3CV, and the stress of TSR 10 is further improved on TSR 10CV (i.e., 15.8 MPa (TSR 3CV), 22.6 MPa (TSR 10CV) and 24.9Mpa (TSR 10) for tensile strength). This indicates that both the natural network structure and the decomposition of nonisoprene components are beneficial to increase the tensile stress of the material. It is worth noting that the contribution of the nonisoprene component to the stress is more obvious than that of the natural network structure. This shows that, for vulcanizates, the decomposition of nonisoprene components after maturation is the key factor for the improvement of mechanical properties. At the same time, after the strain reaches 400%, the stress-strain curves of the three samples have obvious differences in the rising trend. That is, the stress-strain curves of TSR 10 and TSR 10CV show a significant upward trend, while the stress-strain curves of TSR 3CV are still relatively flat. The reason for the above phenomenon may be related to the different effective crosslink density and strain-induced crystallization behavior among the samples. For this reason, the network structure of the vulcanized sample was further fitted and analyzed by the Mooney-Rivlin equation.

The strain-induced crystallization behavior of NR has received extensive attention as an important feature of its comprehensive properties superior to synthetic rubber [37]. The naturally occurring network has been proven to have an important contribution to the generation of the strain-induced crystallization behavior of NR. Furthermore, as for vulcanization process, the chemical networks formed by the introduction of vulcanizing agents also induce strain-induced crystallization behavior [12]. Therefore, in order to have a deeper understanding of the strain-induced crystallization behavior of TSR 10, TSR 10CV and TSR 3CV after vulcanization, the samples were simulated and calculated by using the revised Mooney-Rivlin equation. The specific calculation process is as follows [38,39]:

(9) $$\sigma =2G_{c}+G_{e}\alpha^{-1}F\left(\alpha \right)$$

(10) $$\;F\left(\alpha \right)=\frac{\alpha_{m}}{2}ln\frac{1+\alpha /\alpha_{m}}{1-\alpha /\alpha_{m}}-\frac{1}{\alpha^{2}}$$

(11) $$\frac{\sigma }{\alpha -1/\alpha^{2}}≅2\left(G_{c}+\frac{G_{e}}{\alpha }\right)\left(1+\frac{1}{3}\frac{\alpha^{2}}{\alpha_{m}{}^{2}}\right)\;$$

(12) $$G_{C}=A_{C}V_{C}K_{B}TN_{A}$$

(13) $${\left(\frac{1}{\alpha_{u}}\right)}^{3}=\frac{2G_{C}}{3\alpha_{m}{}^{2}C_{e}}$$

where ơ is the stress at uniaxial tension, α is the elongation at uniaxial tension, $\alpha_{m}$ is the elongation at break, *A_c_* is the correction coefficient of the non-affine network (A_c_ = 0.67), and KB is the Boltzmann constant [40]. T is the absolute temperature, *N_A_* is the Avogadro constant, and $\alpha_{u}$ is the initial strain value of strain-induced crystallization; *G_c_* is the modulus of elasticity contributed by the relative permanent crosslinking point, which represents the degree of chemical crosslinking formed by the end groups of the molecular chain; *G_e_* is the modulus from the entanglements, which represents the degree of the physical entanglement between molecular chains. The values of *G_c_* and *G_e_* are determined by the *y*-axis intercept and the slope of the fitting line. In order to avoid the influence of large strain and finite ductility on the strain-induced crystallization behavior, the deformation range of linear fitting is 0.4 to 0.7 [41]. The specific values after fitting are summarized in Table 4.

**Figure 4:**
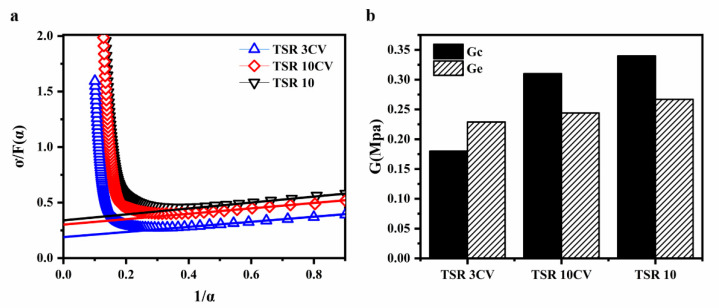
(**a**) Mooney-Rivlin curve of the revised sample; (**b**) *G_c_* and *G_e_* values of the sample.

Figure 4 shows the modified curve of Mooney-Rivlin equation for the three vulcanizated NR samples. Comparing TSR 10CV and TSR 3CV, it can be found that the effective crosslinking density of TSR 10CV has been greatly improved. It is well known that the higher the effective crosslink density, the better the mechanical properties of the material. At the same time, compared with TSR 3CV, the *G_c_* value of TSR 10CV is significantly increased (i.e., 0.32 MPa (TSR 10CV) and 0.18 MPa (TSR 3CV) for *G_c_*), while the *G_e_* value is at the same level. This indicates that the increase in effective crosslinking density of vulcanized rubber due to the decomposition of nonisoprene components is mainly due to the increase in chemical crosslinking network. Furthermore, the increase in effective crosslink density effectively reduces the onset strain ($\alpha_{u}$) of strain-induced crystallization, i.e., the SCI behavior appears earlier. Therefore, the increase in effective crosslink density and the earlier occurrence of strain-induced crystallization are the main reasons for the improved mechanical properties of TSR 10CV. Comparing TSR 10CV and TSR 10, it can be found that since both have undergone the maturation process, they have a similar degree of decomposition of nonisoprene components, thus resulting in approximately the same value of *G_c_* for both. In addition, the *G_e_* value of TRS 10 is higher, which is related to the presence of more natural network structure within it. However, it is worth noting that the magnitude of this improvement is significantly limited (i.e., 0.27 MPa (TSR 10) and 0.24 MPa (TSR 10CV) for *G_e_*). At the same time, the effective crosslink density and au of TSR 10 showed only a small improvement compared to TSR 10CV. This indicates that the naturally occurring network has a certain contribution to the improvement of the mechanical properties of the vulcanizate, but the magnitude of the contribution is relatively small. This is also consistent with the trend exhibited by the stress-strain curves of the two.

**Figure 5:**
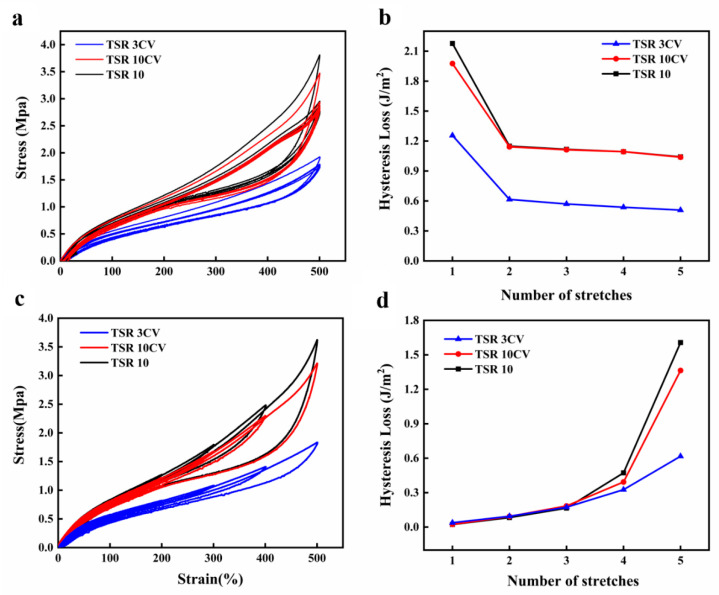
Energy dissipation of NR vulcanizates during tensile cycle progress. (**a**) Cyclic hysteresis curves of NR vulcanizates at 500% fixed strain; (**b**) Hysteresis loss of NR vulcanizates progress at 500% fixed strain; (**c**) Cyclic hysteresis curves of NR vulcanizates at different strain; (**d**) Hysteresis loss of NR vulcanizates progress at different strain.

To further discuss the contribution effect of the two network structures during the tensile process of the specimens, three samples were subjected to cyclic tensile tests. Figure 5a,b shows the cyclic hysteresis curves of three vulcanizated samples at 500% fixed strain, the area of each strain cycle hysteresis curve in Figure 5a is calculated and described as a hysteresis loss point in Figure 5b. As shown in Figure 5b, the energy dissipation of the sample gradually decreases with the increase in the number of cycles, showing a typical “Mullins effect” [42]. This phenomenon is mainly related to molecular chain slippage, disentanglement, and network structure disruption, while strain-induced crystallization is reversible during this process [43]. It can be found that TSR 10 has the greatest reduction in energy loss during the first and second stretches (i.e., 1.0 MPa for the difference between the two energy losses before and after), which is due to there being more naturally occurring networks in TSR 10, and this physical network structure is more fragile than chemically cross-linked networks, so it will be destroyed under large strains, acting as a “sacrificial bond” [44]. While TSR 10CV and TSR 3CV decrease less in energy loss due to the lower number of naturally occurring networks present (i.e., 0.8 MPa (TSR 10CV) and 0.7 MPa (TSR 3CV) for the difference between the two energy losses before and after). However, it is worth noting that in the comparison of the second, third and fourth energy losses that weaken the effect of the physical network, it can be found that TSR 10 and TSR 10CV have significantly higher energy losses than TSR 3CV. This phenomenon may be related to the molecular chains of different chain lengths in the internal network, which is explained by the energy loss of the samples under superimposed strains of 100–500%.

Figure 5c,d shows the cyclic hysteresis curves of three vulcanizated samples at different strain, the area of each strain cycle hysteresis curve in Figure 5c is calculated and described as a hysteresis loss point in Figure 5d As for Figure 5d the TSR 10, TSR 10CV, and TSR 3CV samples have similar hysteresis losses at low strains of 100–300%, and the hysteresis loss values of the samples show a significant upward trend when the strain exceeds 300%. In particular, the hysteresis loss values of TRS 10 and TRS 10CV exhibit a significant and similar upward trend at 400% and 500% strain, while this trend of TSR 3CV sample is weaker. The above phenomenon is mainly caused by the stress concentration phenomenon determined by the finite ductility. According to the theory of rubber elasticity [45,46], the extension of molecular segments is limited, and the strain at the breaking point depends on the molecular weight (Mc) [47]. Therefore, higher crosslink density results in lower Mc between network points and earlier initial strain of break. At the same time, the non-Gaussian chain model and the non-Gaussian tube model confirmed a sharp increase in tensile stress near the fracture strain [48,49,50]. Therefore, the molecular segments between the branch points of the three samples are far from their critical value in the low strain region of 100–300%, which result in only a small increase in stress. However, when the strain increased to 300–500%, the decomposition products of nonisoprene components promoted the formation of chemical cross-linking networks and cross-linking points, which significantly shortened the length of the molecular chain between the cross-linking points, resulting in stronger stress concentration and hysteresis loss during stretching of TSR 10 and TSR 10CV samples. Furthermore, the hysteresis loss value of TSR 10 is slightly higher than that of TSR 10CV, which demonstrates the contribution of the naturally generated network. Clearly, the naturally occurring network is much weaker than the chemically cross-linked network in terms of the mechanical contribution of the vulcanized samples. This naturally occurring network structure exists in the form of sacrificial bonds. Although its contribution is weak, it cannot be ignored.

### 3.3. Compression Heat Generation and Fatigue Life of NR Vulcanizates

**Figure 6:**
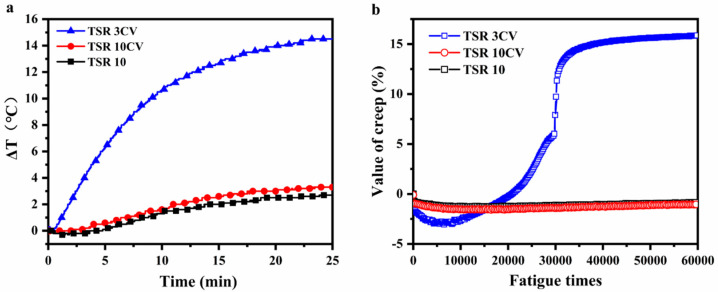
(**a**) Temperature rise curve of vulcanizates; (**b**) Creep and fatigue life curve of vulcanizates.

The heat generated in the movement of NR is difficult to release due to its weak thermal conductivity. Such heat is absorbed in only two ways. One is absorbed via the network structure of NR and it causes irreversible deformation and creep of the network structure. Another converted into internal temperature rise. Therefore, the dynamic mechanical properties of materials can be reflected by testing the changes of temperature and network structure during the dynamic loading process. The dynamic mechanical properties of the three vulcanized NRs are shown in Figure 6. TSR 10 and TSR 10CV have a similar level of temperature rise, and the fatigue life are both greater than 60,000 times. Additionally, the temperature rise of TSR 3CV is much higher than that of TSR 10CV, and the fatigue life is only 30,000 times; this phenomenon is related with the conformational entropy change of the samples. In order to analyze the influence of the network structure difference on thermogenesis, it is necessary to study the dynamic changes of the network structure under stress. The thermodynamic statistical method is used to calculate the entropy and creep of the samples under dynamic load. The specific calculation process is as follows [51]:

(14) $$N=\frac{M_{c}}{M_{s}}$$

(15) $$G_{e}=\frac{1}{4*6^{\frac{1}{2}}}K_{B}Tn_{s}$$

(16) $$d_{0}=l_{S}ne^{\frac{1}{2}}$$

(17) $$\Delta S=\frac{-3K_{B}V_{C}N_{A}}{2Nl_{S}}(\frac{N^{2}}{n_{s}d_{0}{}^{2}}\alpha +d_{0}{}^{2}\alpha^{-\frac{1}{2}}-\frac{1}{6}Nl_{s}{}^{2}ln\alpha -\frac{N^{2}}{n_{s}d_{0}{}^{2}}-d_{0}{}^{2}$$

where *N* is the average number of chain segments between two adjacent cross-linking points, *M_s_* is the molar mass of a single chain segment in NR, *M_s_* = 105 g/mol, *d*_0_ is the fluctuation range of the chain segment, *l_s_* is the Kuhn segment (generally take 0.76 nm in NR), *n_s_* is the number density of NR segments, *n_s_* = 5.46 nm^−3^; *n_e_* is the number of segments between two adjacent entanglement points [52,53].

The temperature rise, fatigue life and related network structure parameters of three vulcanizated samples are summarized in Table 5. It can be found that the dynamic mechanical properties of TSR 3CV are the worst in these three samples, which is due to the lowest content of chemical cross-linked networks and naturally occurring networks inside the TSR 3CV sample. This results in the molecular chain moving more easily in its internal structure, thus generating more energy during dynamic loading process. As for TSR 10CV, its dynamic mechanical properties have been significantly improved, which indicates that the cross-linked points can restrict the motion of rubber chains, and have the impeding and “pinning” effect during the deformation process of the rubber chain [51]. Tubular model theory also proves this. According to the tubular model theory, the smaller the motion radius (*d*_0_) of the molecular chain led to the lower energy generated during the loading process. Therefore, more chemical crosslinking networks and entanglement points in TSR 10CV can impose more restrictions on the movement of molecular chains, thus significantly reducing the value of *d*_0_, which effectively reduces the entropy change of TSR 10CV under dynamic loads. As for TSR 10 sample, the dynamic mechanical properties have been further improved. This is related to the abundant naturally occurring network act as a sacrificial bond to dissipate energy, such sacrificial bonds replace the chemical bonds and are broken first. The above results show that the naturally occurring network and the decomposition of nonisoprene components are beneficial to improve the dynamic mechanical properties of the materials after maturation. However, from the effect of contribution, the decomposition of nonisoprene components is still more significant.

## 4. Conclusions

Three types of NR, i.e., TSR 10, TSR 10CV and TSR 3CV, were prepared and used to study the respective effects of naturally occurring network and nonisoprene degradation on the mechanical properties of natural rubber. The results show that the structuring of the natural network during maturation causes a significant increase in the initial plasticity value, Mooney viscosity and gel content, while the degradation of nonisoprene components has little effect on the structure and properties of raw rubber. After preparation into pure rubber formula vulcanizate, it was found that the decomposition products of nonisoprene components can act as a vulcanization promoter, which significantly increases the effective crosslink density of the material, especially in terms of chemical crosslinking network. This results in a significant improvement in mechanical properties such as tensile strength and hardness. At the same time, more chemical cross-linking points will constrain the movement of molecular chains under dynamic loading, thereby reducing the entropy change to endow the material with better dynamic mechanical properties. The naturally occurring network structure mainly uses the form of sacrificial bonds to assist in improving the overall performance of the material, and the contribution effect is relatively weak, and it mainly has a synergistic effect.

## Figures and Tables

**Table 1:** Formulation of NR compounds.

Chemical	Quantity (Phr)
Natural rubber	100.0
Stearic acids	0.5
Accelerator M	0.5
ZnO	6.0
Sulfur	3.5

**Table 2:** The different contents of nonisoprene components in TSR 10, TSR 10CV, TSR 3CV.

Sample	Nitrogen Content ^a^ (wt.%)	C_14_^b^ (wt.%)	C_16_ ^b^ (wt.%)	C_17_ ^b^ (wt.%)	C_18_H_32_O_2_ ^b^ (wt.%)	C_18_H_34_O_2_ ^b^ (wt.%)	C_18_H_36_O_2_ ^b^ (wt.%)	C_20_ ^b^ (wt.%)	Total Fatty Acid ^b^ (wt.%)
TSR 3CV	0.38	-	0.18	-	0.78	0.43	0.31	0.03	1.73
TSR 10CV	0.15	0.04	0.35	-	0.06	0.07	0.23	0.09	0.84
TSR 10	0.15	0.06	0.38	-	0.04	0.05	0.21	0.07	0.81

^a^ Values obtained from Nitrogen content tests; ^b^ Results obtained from Gas chromatography.

**Table 3:** Structure parameters of un-vulcanized NR for TSR 10, TSR 10CV and TSR 3CV.

Sample	Gel Content (wt.%)	Initial Plasticity (*P*_0_)	Mooney Viscosity *ML* (1 + 4)	*PRI*	Δ*M*	Δ*P*
TSR 3CV	19.8	42.5	89.8	75.6	4.5	3.0
TSR 10CV	20.2	44.0	91.3	55.6	7.4	4.5
TSR 10	33.6	64.0	108.6	53.9	15.3	13.0

*PRI*, plasticity retention index. Δ*P*, change in plasticity. Δ*M*, change in *ML* (1 + 4).

**Table 4:** Rubber network structure parameters.

Sample	*G_c_*, Mpa	*G_e_*, Mpa	$a_{u}\;$	*V_c_*, 10^−4^mol·cm^−3^
TSR 10	0.34	0.27	4.33	2.87
TSR 10CV	0.32	0.24	4.35	2.61
TSR 3CV	0.18	0.23	5.75	1.52

**Table 5:** Temperature rise, fatigue life and related network structure parameters of NR vulcanizates.

Sample	*N*	*d*_0_, nm	*n_e_*	∆*S, * J·cm^3^·K^−1^	∆*T,* °C	Fatigue, Million Times
TSR 3CV	57.75	2.30	9.12	5.79	14.5	3.01
TSR 10CV	33.53	2.25	8.74	2.12	3.3	>6
TSR 10	29.53	2.12	7.77	2.09	2.7	>6

## Data Availability

Not applicable.

## References

[1] Tanaka Y. (2001). Structural Characterization of Natural Polyisoprenes: Solve the Mystery of Natural Rubber Based on Structural Study. Rubber Chem. Technol..

[2] Park H.S., Woo C.S. (2007). Mechanical Properties Evaluation of Natural and Synthetic Rubber. Elastomers Compos..

[3] Svenningsson L., Sparrman T., Bialik E., Bernin D., Nordstierna L. (2019). Molecular orientation distribution of regenerated cellulose fibers investigated with rotor synchronized solid state NMR spectroscopy. Cellulose.

[4] Chen P., Terenzi C., Furó I., Berglund L.A., Wohlert J. (2019). Quantifying Localized Macromolecular Dynamics within Hydrated Cellulose Fibril Aggregates. Macromolecules.

[5] Sainumsai W., Toki S., Amnuaypornsri S., Nimpaiboon A., Sakdapipanich J., Rong L., Hsiao B.S., Suchiva K. (2017). Dependence of The Onset of Strain-Induced Crystallization of Natural Rubber and Its Synthetic Analogue on Crosslink and Entanglement by Using Synchrotron X-Ray. Rubber Chem. Technol..

[6] Ikeda Y., Yasuda Y., Hijikata K., Tosaka M., Kohjiya S. (2008). Comparative Study on Strain-Induced Crystallization Behavior of Peroxide Cross-Linked and Sulfur Cross-Linked Natural Rubber. Macromolecules.

[7] Ortiz C., Boyce M. (2008). Materials science. Bioinspired structural materials. Science.

[8] Bottier C., Nawrot R. (2020). Biochemical composition of Hevea brasiliensis latex: A focus on the protein, lipid, carbohydrate and mineral contents. Latex, Laticifers and Their Molecular Components: From Functions to Possible Applications.

[9] Mcmahan C., Kostyal D., Lhamo D., Cornish K. (2015). Protein influences on guayule and Hevea natural rubber sol and gel. J. Appl. Polym. Sci..

[10] Xu L., Cheng H., Luo M., Wei Q., Jing Z. (2015). A rheological study on non-rubber component networks in natural rubber. RSC Adv..

[11] Liu J., Wu S., Tang Z., Lin T., Guo B., Huang G. (2015). New evidence disclosed for networking in natural rubber by dielectric relaxation spectroscopy. Soft Matter.

[12] Toki S., Hsiao B.S., Amnuaypornsri S., Sakdapipanich J. (2009). New insights into the relationship between network structure and strain-induced crystallization in un-vulcanized and vulcanized natural rubber by synchrotron X-ray diffraction. Polymer.

[13] Wang Y., Liu H., Yu H., Zhao P., Wang Q., Liao L., Luo M., Zheng T., Liao S., Peng Z. (2022). New insight into naturally occurring network and entanglements induced strain behavior of vulcanized natural rubber. Polymer.

[14] Ping-Yue W., Yong-Zhou W., Bei-Long Z., Hong-Hai H. (2012). Effect of non-rubber substances on vulcanization kinetics of natural rubber. J. Appl. Polym. Sci..

[15] Wei Y.C., Liu G.X., Zhang H.F., Zhao F., Liao S. (2019). Non-rubber components tuning mechanical properties of natural rubber from vulcanization kinetics. Polymer.

[16] Nun-Anan P., Wisunthorn S., Pichaiyut S., Vennemann N., Nakason C. (2018). Novel approach to determine non-rubber content in Hevea brasiliensis: Influence of clone variation on properties of un-vulcanized natural rubber. Ind. Crops Prod..

[17] Moreno R.M.B., Ferreira M., Gonçalves P.D.S., Mattoso L.H.C. (2005). Technological properties of latex and natural rubber of Hevea brasiliensis clones. Sci. Agric..

[18] Galiani P.D., Martins M.A., Gon Alves P., Mcmahan C.M., Mattoso L. (2011). Seasonal and clonal variations in technological and thermal properties of raw Hevea natural rubber. J. Appl. Polym. Sci..

[19] Bonfils F., Doumbia A., Char C., Beuve J.S. (2010). Evolution in the natural rubber native structure and plasticity retention index from the first tapping of clonal trees. J. Appl. Polym. Sci..

[20] Chen X., Zhang H. (2021). Insight on natural rubber’s relationship with coagulation methods and some of its properties during storage. J. Appl. Polym. Sci..

[21] Intapun J., Sainte-Beuve J., Bonfils F., Tanrattanakul V., Dubreucq E., Vaysse L. (2010). Effect of microorganisms during the initial coagulum maturation of Hevea natural rubber. J. Appl. Polym. Sci..

[22] Salomez M., Subileau M., Intapun J., Bonfils F., Sainte-Beuve J., Vaysse L., Dubreucq E. (2015). Micro-organisms in latex and natural rubber coagula of Hevea brasiliensis and their impact on rubber composition, structure and properties. J. Appl. Microbiol..

[23] Salomez M., Subileau M., Vallaeys T., Santoni S., Bonfils F., Sainte-Beuve J.M., Intapun J., Granet F.O., Vaysse L., Dubreucq É. (2018). Microbial communities in natural rubber coagula during maturation: Impacts on technological properties of dry natural rubber. J. Appl. Microbiol..

[24] Zhong J.P., Li C.P., Ll S.D., Kong L.X., She X.D. (2009). Study on the Properties of Natural Rubber during Maturation. J. Polym. Mater..

[25] Fri P.S., Nkeng G.E., Ehabe E.E. (2007). Effect of natural coagula maturation on the processability, cure, and mechanical properties of unfilled vulcanizates of Hevea natural rubber. J. Appl. Polym. Sci..

[26] Ehabe E., Roux Y.L., Ngolemasango F., Bonfils F., Nkeng G., Nkouonkam B., Sainte-Beuve J., Gobina M.S. (2010). Effect of maturation on the bulk viscosity and molecular chain length of cuplump natural rubber. J. Appl. Polym. Sci..

[27] Qi N.L., Li P.W., Zeng X.H., Huang H.H., Yang Z.M., Gong X. (2013). Comparison of Kjeldahl and the Elemental Analysis Methods for Determination of Nitrogen Content in Raw Natural Rubber. Adv. Mater. Res..

[28] Arrondo J.L.R., Muga A., Castresana J., Goni F.M. (1993). Quantitative studies of the structure of proteins in solution by fourier-transform infrared spectroscopy. Prog. Biophys. Mol. Biol..

[29] Vennemann N., Bökamp K., Bröker D. (2010). Crosslink Density of Peroxide Cured TPV. Macromol. Symp..

[30] Flory P.J. (1952). Molecular Size Distribution in Three Dimensional Polymers. VI. Branched Polymers Containing A—R—Bf-1 Type Units. J. Am. Chem. Soc..

[31] Shah A.A., Hasan F., Shah Z., Kanwal N., Zeb S. (2013). Biodegradation of natural and synthetic rubbers: A review. Int. Biodeterior. Biodegrad..

[32] Tarachiwin L., Sakdapipanich J., Ute K., Kitayama T., Tanaka Y. (2005). Structural characterization of alpha-terminal group of natural rubber. 2. Decomposition of branch-points by phospholipase and chemical treatments. Biomacromolecules.

[33] Wei Y.C., Liu G.X., Zhang L., Zhao F., Liao S., Luo M.C. (2020). Exploring the unique characteristics of natural rubber induced by coordination interaction between proteins and Zn 2+. Polymer.

[34] Chukwu M.N., Madufor I.C., Ayo M.D., Ekebafe L.O. (2011). Effect of Stearic Acid Level on the Physical Properties of Natural Rubber Vulcanisate. Pac. J. Sci. Technol..

[35] Poh B.T., Tan E.K. (2010). Mooney scorch time and cure index of epoxidized natural rubber in presence of sodium carbonate. J. Appl. Polym. Sci..

[36] Amnuaypornsri S., Sakdapipanich J., Tanaka Y. (2010). Highly purified natural rubber by saponificaion of latex: Analysis of green and cured properties. J. Appl. Polym. Sci..

[37] Che J., Burger C., Toki S., Rong L., Hsiao B.S., Amnuaypornsri S., Sakdapipanich J. (2013). Crystal and Crystallites Structure of Natural Rubber and Synthetic cis- 1,4-Polyisoprene by a New Two Dimensional Wide Angle Xray Diffraction Simulation Method. I. Strain-Induced Crystallization. Macromolecules.

[38] Mooney M. (1940). A Theory of Large Elastic Deformation. J. Appl. Phys..

[39] Treloar L. (1940). Theory of Large Elastic Deformations. Nature.

[40] Nie Y., Wang B., Huang G., Qu L., Zhang P., Weng G., Wu J. (2010). Relationship between the material properties and fatigue crack-growth characteristics of natural rubber filled with different carbon blacks. J. Appl. Polym. Sci..

[41] Schl Gl S., Trutschel M.L., Chassé W., Riess G., Saalw Chter K. (2014). Entanglement Effects in Elastomers: Macroscopic vs. Microscopic Properties. Macromolecules.

[42] Diani J., Fayolle B., Gilormini P. (2009). A review on the Mullins effect. Eur. Polym. J..

[43] Trabelsi S., Albouy P.A., Rault J. (2003). Effective Local Deformation in Stretched Filled Rubber. Macromolecules.

[44] Liu J., Tang Z., Huang J., Guo B., Huang G. (2016). Promoted strain-induced-crystallization in synthetic cis-1,4-polyisoprene via constructing sacrificial bonds. Polymer.

[45] Edwards S.F. (1977). The theory of rubber elasticity. Br. Polym. J..

[46] Boyce M.C., Arruda E.M. (2000). Constitutive Models of Rubber Elasticity: A Review. Rubber Chem. Technol..

[47] Miehe C., Goektepe S., Lulei F. (2004). A micro-macro approach to rubber-like materials—Part I: The non-affine micro-sphere model of rubber elasticity. J. Mech. Phys. Solids.

[48] Gent A.N. (2012). A New Constitutive Relation for Rubber. Rubber Chem. Technol..

[49] Edwards S.F., Vilgis T.A. (1988). The tube model theory of rubber elasticity. Rep. Prog. Phys..

[50] Klüppel M., Menge H., Schmidt H., Schneider H., Schuster R.H. (2001). Influence of Preparation Conditions on Network Parameters of Sulfur-Cured Natural Rubber. Macromolecules.

[51] Zhan Y.H., Wei Y.C., Zhang H.F., Luo M.C., Zheng T.T., Liao S. (2020). Analysis of the thermogenesis mechanism of natural rubber under high speed strain. Polym. Adv. Technol..

[52] Aharoni S.M. (1986). Correlations between chain parameters and the plateau modulus of polymers. Macromolecules.

[53] Klüppel M., Schramm J. (2000). A generalized tube model of rubber elasticity and stress softening of filler reinforced elastomer systems. Macromol. Theory Simul..

